# Supplementary material for: The genome sequence of the European mole, Talpa europaea Linnaeus, 1758
Source: Wellcome Open Res. 2025 Feb 24;10:98. [Version 1] doi: 10.12688/wellcomeopenres.23759.1 (PMC11909498; doi:10.12688/wellcomeopenres.23759.1)
Supplement: Peer review history

# Reviewer report: Reviewer response for version 1

**DOI:** 10.21956/wellcomeopenres.26207.r120540

**Published:** 2025-03-17

**Authors:** Phillip A Morin

**Affiliations:** 1 National Marine Fisheries Service, Southwest Fisheries Science Center, La Jolla, CA, USA

This report presents a new genome assembly from a European mole, *Talpa europaea*. It was assembled using PacBio HiFi and Illumina Hi-C data. The assembly meets the VGP assembly quality standards as adopted by the Earth BioGenomes Project. 

 In the Background section, the sentence starting with "Whilst it is listed as ‘Least Concern’ globally..." needs a reference for where it's listed as 'Least Concern', e.g., (IUCN redlist, consulted Feb. 2025).

 The methodologies appear sound and suitable.

Are sufficient details of methods and materials provided to allow replication by others?

Yes

Is the rationale for creating the dataset(s) clearly described?

Yes

Are the datasets clearly presented in a useable and accessible format?

Yes

Are the protocols appropriate and is the work technically sound?

Yes

Reviewer Expertise:

Population genetics and genomics of cetaceans

I confirm that I have read this submission and believe that I have an appropriate level of expertise to confirm that it is of an acceptable scientific standard.

# Reviewer report: Reviewer response for version 1

**DOI:** 10.21956/wellcomeopenres.26207.r120539

**Published:** 2025-03-14

**Authors:** Frédéric Delsuc

**Affiliations:** 1 Universite de Montpellier, Montpellier, Occitanie, France

This data note reports on the chromosome-scale genome sequence of the European mole ( *Talpa europaea*) as part of the Darwin Tree of Life initiative. The technical aspects of the genome sequencing follow the standards established by the Darwin Tree of Life Consortium and are therefore perfectly sound and well described.

 However, I think that the rationale for sequencing this particular genome could be more clearly justified. Indeed, not much is said about the potential applications and benefits that access to this genome will have for the evolutionary biologist and conservationist communities. Moreover, the background on previous molecular studies of talpids, including the European mole, is incomplete, with no mention of the recent discovery of a new and previously undescribed species ( *Talpa aquitania*) within the presumed geographical distribution of Iberian and European moles in southwestern France and northern Spain (Nicolas et al., 2017a,b). This new species has been discovered through both molecular and morphological studies and the distributions of the two mole species occurring in western Europe have been reassessed accordingly (Nicolas et al. 2021). I think that this information is important to mention in this genome paper even though the representative specimen of *Talpa europaea* sequenced here is from England. 

**Typo:**

- In the 5th paragraph of the background section, Talpidae is misspelled as Talipidae.

Are sufficient details of methods and materials provided to allow replication by others?

Yes

Is the rationale for creating the dataset(s) clearly described?

Partly

Are the datasets clearly presented in a useable and accessible format?

Yes

Are the protocols appropriate and is the work technically sound?

Yes

Reviewer Expertise:

Mammalian phylogenomics and comparative genomics.

I confirm that I have read this submission and believe that I have an appropriate level of expertise to confirm that it is of an acceptable scientific standard.

## References

[1] : Molecular data and ecological niche modelling reveal the evolutionary history of the common and Iberian moles (Talpidae) in Europe. *Zoologica Scripta* .2017;46(1) : 10.1111/zsc.12189 12-26 10.1111/zsc.12189

[2] : Talpa aquitania sp. nov. (Talpidae, Soricomorpha), a new mole species from SW France and N Spain. *Mammalia* .2017;81(6) : 10.1515/mammalia-2017-0057 641-642 10.1515/mammalia-2017-0057

[3] : New data on the distribution of the two mole species Talpa aquitania Nicolas, Matinez-Vargas & Hugot, 2017 and T. europaea Linnaeus, 1758 in France based on museum and newly collected specimens. *Zoosystema* .2021;43(24) : 10.5252/zoosystema2021v43a24 10.5252/zoosystema2021v43a24

# Reviewer report: Reviewer response for version 1

**DOI:** 10.21956/wellcomeopenres.26207.r119460

**Published:** 2025-03-14

**Authors:** Sadık Demirtaş

**Affiliations:** 1 Department of Molecular Biology and Genetics, Faculty of Science, Ondokuz Mayis University, Samsun, Turkey

The taxonomy, distribution and biology of the species are sufficiently reported. The references are sufficient and contemporary. Both the sequencing and assembly statistics are consistent and compatible. The software programs,figures and tables used in the article are sufficiently understandable. I believe that this study will be a good and reliable reference for future studies with other *Talpa* spp.

Are sufficient details of methods and materials provided to allow replication by others?

Yes

Is the rationale for creating the dataset(s) clearly described?

Yes

Are the datasets clearly presented in a useable and accessible format?

Yes

Are the protocols appropriate and is the work technically sound?

Yes

Reviewer Expertise:

Animal taxonomy, animal genetics and molecular phylogeny

I confirm that I have read this submission and believe that I have an appropriate level of expertise to confirm that it is of an acceptable scientific standard.
